# Neuroprotective effects of protocatechuic aldehyde through PLK2/p-GSK3β/Nrf2 signaling pathway in both *in vivo* and *in vitro* models of Parkinson's disease

**DOI:** 10.18632/aging.102394

**Published:** 2019-11-06

**Authors:** Chao Guo, Junrong Zhu, Jingwen Wang, Jialin Duan, Shanbo Ma, Ying Yin, Wei Quan, Wei Zhang, Yue Guan, Yi Ding, Aidong Wen, Yingdong Zhang

**Affiliations:** 1 School of Basic Medicine and Clinical Pharmacy, China Pharmaceutical University, Nanjing 211198, China; 2 Department of Pharmacy, Xijing Hospital, Fourth Military Medical University, Xi’an 710032, China; 3 Nanjing First Hospital, China Pharmaceutical University, Nanjing 211198, China; 4 Mental Health Center, Department of Medicine, Xi’an Jiaotong University, Xi’an 710199, China

**Keywords:** protocatechuic aldehyde, reactive oxygen species, mitochondrial dysfunction, Parkinson's disease, PLK2-Nrf2 pathway

## Abstract

Mitochondrial dysfunction and oxidative damage are closely related to the pathogenesis of Parkinson's disease (PD). The pharmacological mechanism of protocatechuic aldehyde (PCA) for PD treatment have retained unclear. The purposes of the present study were to clarify the neuroprotective effects of post-treatment of PCA for PD treatment by mitigating mitochondrial dysfunction and oxidative damage, and to further determine whether its effects were mediated by the polo-like kinase 2/phosphorylated glycogen synthase kinase 3 β/nuclear factor erythroid-2-related factor 2 (PLK2/p-GSK3β/Nrf2) pathways. We found that PCA improved 1-methyl-4-phenyl-1, 2, 3, 6-tetrahydropyridine (MPTP)-induced behavioral deficits and dopaminergic cell loss. Moreover, PCA increased the expressions of PLK2, p-GSK3β and Nrf2, following the decrease of α-synuclein (α-Syn) in MPTP-intoxicated mice. Cell viability was increased and the apoptosis rate was reduced by PCA in 1-methyl-4-phenylpyridinium iodide (MPP^+^)-incubated cells. Mitochondrial membrane potential (MMP), mitochondrial complex I activity and reactive oxygen species (ROS) levels in MPP^+^-incubated cells were also ameliorated by treatment with PCA. The neuroprotective effects of PCA were abolished by inhibition or knockdown of PLK2, whereas overexpression of PLK2 strengthened the protection of PCA. Furthermore, GSK3β and Nrf2 were involved in PCA-induced protection. These results indicated that PCA has therapeutic effects on PD by the PLK2/p-GSK3β/Nrf2 pathway.

## INTRODUCTION

Parkinson's disease (PD) is one of the most common neurodegenerative diseases, affecting approximately 2% of the world's population over 60 years of age [1]. Clinically, PD is characterized by motor symptoms, including muscle rigidity, static tremor, bradykinesia, and postural instability, as well as non-motor symptoms including cognitive impairment, olfactory dysfunction, psychiatric symptoms, sleep disorders and autonomic dysfunction. The pathological feature of PD is the progressive degeneration of dopaminergic neurons in the substantia nigra (SN), resulting in a deficiency of dopamine (DA) in the striatum [2]. Drugs now in clinical use, including levodopa, catechol-O-methyltransferase inhibitors, monoamine oxidase type B inhibitors, and serotonin agonists, mainly improve the symptoms by supplementing the lack of dopamine, but do not delay disease progression [3]. Moreover, the long-term usage of levodopa produces side-effects like motor fluctuations and dyskinesias [4]. Thus, there is still an urgent need to develop an anti-PD agent which could not only alleviate PD symptoms, but also produce neuroprotective effects.

Although the precise pathogenesis of PD still remains elusive, several studies demonstrate that mitochondrial dysfunction and oxidative damage play the important roles in PD pathogenesis [5–7]. It has been revealed that 25–30% of PD patients have significant deficiency of mitochondrial complex I and increased reactive oxygen species (ROS) levels [8]. Mitochondrial dysfunction in the pathogenesis of PD is caused by a defect within complex I of the electron transport chain [9]. The defects in the respiratory chain which result in the disruption of electron transfer lead to oxidative stress [10]. Oxidative stress reflects an imbalance between the overproduction of ROS and the ability of the body to reject the toxic effects through antioxidant defense systems such as glutathione, superoxide dismutase (SOD), catalase, and glutathione peroxidase (GPX). Previous studies have reported that the antioxidant defense system could be affected, resulting in a large quantity of related ROS in the brain of PD mouse models [11]. Therefore, reducing or blocking oxidative stress represents a viable therapeutic method for PD.

Nuclear factor erythroid-2-related factor 2 (Nrf2) is a member of the key regulators of cytoprotective and detoxification genes to resist oxidative stress. When exposed to oxidative stress, Nrf2 dissociates from keap1 and is prompted to transfer into the cell nucleus and then binds to the antioxidant response element (ARE) to induce a battery of antioxidant and phase II detoxification enzymes, such as NADPH quinone oxidoreductase1 (NQO1) and hemeoxygenase-1 (HO-1). Many reports have suggested that Nrf2-mediated antioxidant shortage may exert a vital role in the oxidative stress commonly associated with PD [12–14]. In postmortem brain tissue from PD patients, the expressions of NQO1 and HO-1 are up-regulated, which indicate a neuroprotective response mediated by Nrf2 activation [15, 16]. Moreover, studies have demonstrated that Nrf2-deficient mice were more sensitive to MPTP-induced toxicity, whereas Nrf2 overexpression may exert the neuroprotection [17–19]. Remarkably, recent studies have also reported that the expression of polo-like kinase 2 (PLK2) is responsive to oxidative stress, which mediates antioxidant signaling by phosphorylating glycogen synthase kinase 3β (GSK3β), thereby promoting the nuclear translocation of Nrf2 [20]. A transgenic analysis further revealed that PLK2 is highly expressed and essential for the survival of cells with mitochondrial dysfunction and increased oxidative stress [21]. The findings suggested that modulating PLK2 activity is a new therapeutic target in PD.

Protocatechuic aldehyde (PCA) is a phenolic acid derived from the roots of the traditional Chinese herb *Salvia miltiorrhiza.* Our previous research reported that PCA has significant neuroprotection on cerebral ischemia reperfusion-induced oxidative injury [22]. Notably, PCA was reported to have potential antioxidative effects through DJ-1 in SH-SY5Y cells, a PD-related gene [23]. Further evidence showed that pre-treatment with PCA can protect dopaminergic neurons against neurotoxin-induced damage both *in vitro* and *in vivo* [24]. These results strongly implied that PCA may be a potential agent for treating PD. However, the neuroprotective effects of post-treatment of PCA and its pharmacological mechanisms against PD-induced injury have remained undefined. To address the issue, this study was designed to identify the molecular mechanism of PCA against PD injury in cell and mouse models and further investigate whether its effects were involved in PLK2-Nrf2 pathway.

## RESULTS

### PCA improved behavioral deficits in MPTP-induced mice

**Figure 1:**
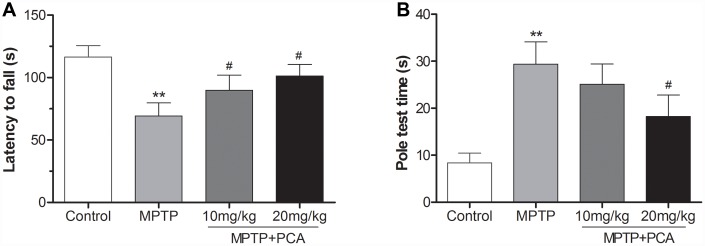
**PCA improved behavioral deficits in MPTP- intoxicated mice.** (**A**) Rotarod test in each group. (**B**) Pole test in each group. Data were expressed as mean ± S.D., n = 12; ^**^P< 0.01*vs.* control group, ^#^*P*< 0.05 *vs.* MPTP group.

To investigate the effects of PCA on motor function, the rotarod and pole tests were conducted in our study. As shown in Figure 1A, the rotarod test showed that mice in the MPTP group stayed on the rod for a shorter time than the controls. However, 10 and 20 mg/kg PCA significantly prolonged their duration on the rod. Furthermore, the pole test (Figure 1B) showed that MPTP significantly prolonged the total time for climbing down the pole compared with controls, whereas post-treatment with 20 mg/kg PCA significantly promoted MPTP-intoxicated mice to spend a shorter time climbing down the pole. The dosage of 10 mg/kg of PCA showed a decreased trend for the time compared with the MPTP group, which did not reach statistical significance. These results suggested that post-treatment with PCA could effectively improve the behavioral deficits in the mouse model of PD.

### PCA attenuated dopaminergic neuronal loss in MPTP-induced mice

**Figure 2:**
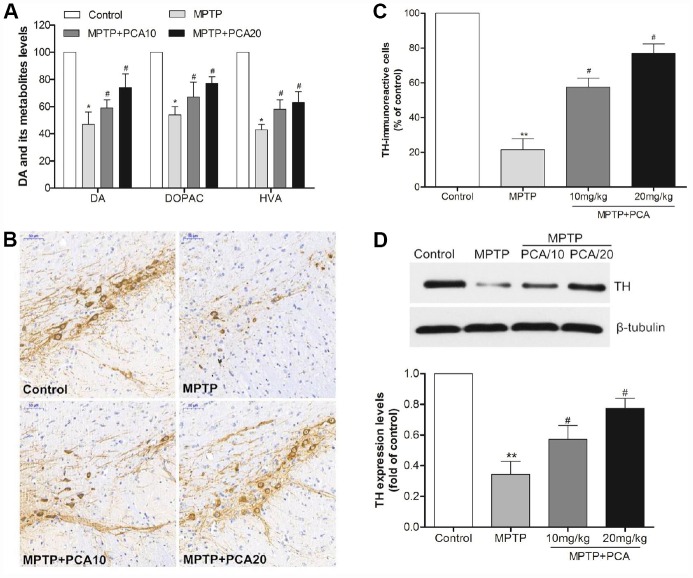
**PCA alleviated dopaminergic neuronal loss in MPTP-intoxicated mice.** (**A**) The levels of dopamine, DOPAC and HVA in the striatum were measured by HPLC. (**B**–**C**) Brain sections were immunostained for TH immunoreactivity in SN and TH positive cells were quantified. Scale bar, 50 μm. (**D**) Representative western blot bands and quantification of TH in each group. Data were expressed as mean ± S.D., n = 6; ^**^P< 0.01,^*^P< 0.05 *vs.* control group; ^#^*P*< 0.05 *vs.* MPTP group.

**Figure 3:**
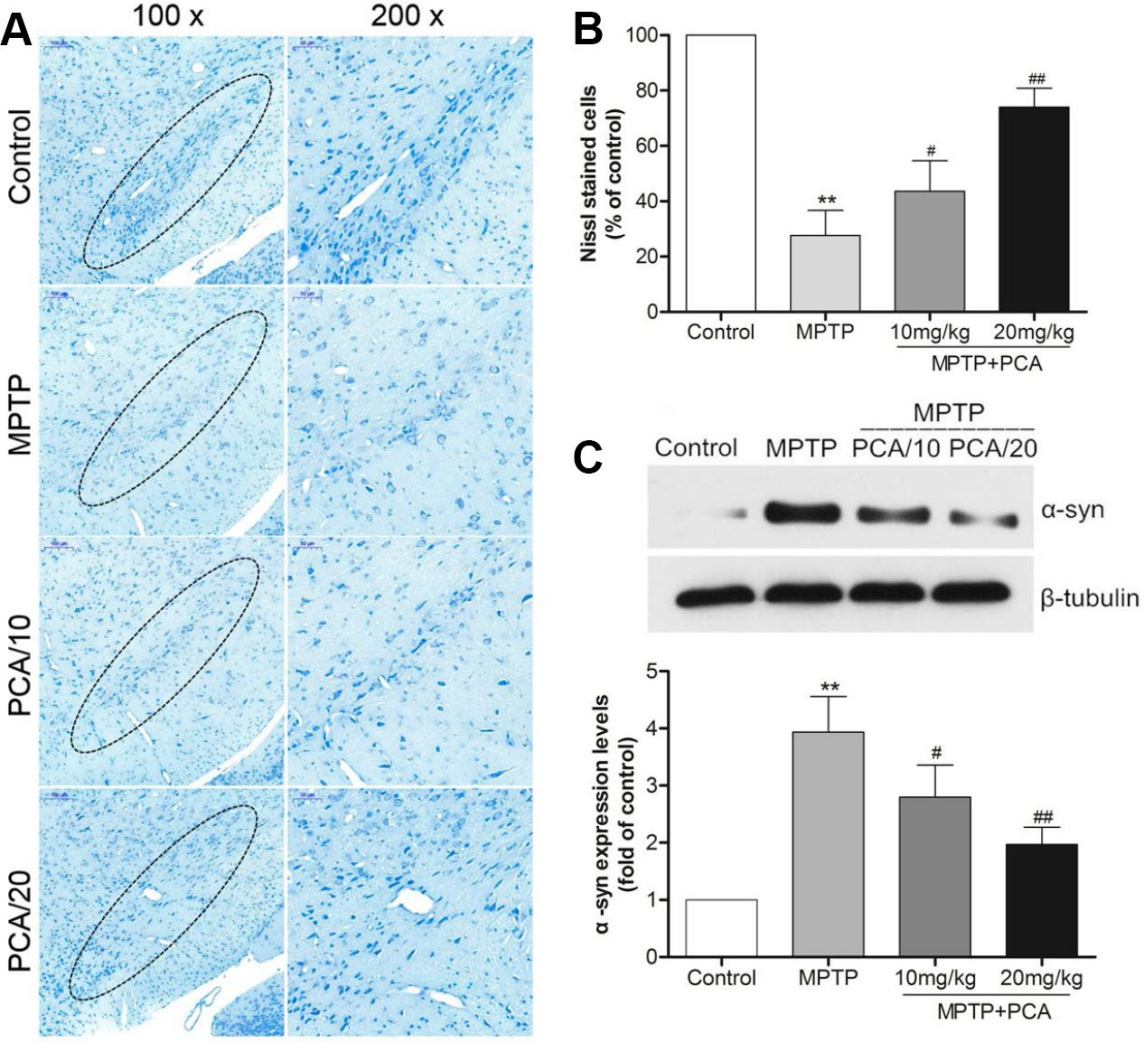
**PCA inhibited neuronal degeneration in MPTP-intoxicated mice.** (**A**) Nissl staining in SN. (**B**) Nissl-positive cells were quantified. (**C**) Representative western blot bands and quantification of α-Syn levels in each group. Scale bar, 50 μm. Data were expressed as mean ± S.D., n = 6; ^*^P< 0.01*vs.* control group, ^#^*P*< 0.05 *vs.* MPTP group.

To evaluate the effects of PCA on MPTP-induced neurotoxicity, we then performed neurochemical analysis with striatal tissue using HPLC analysis. The results (Figure 2A) showed that MPTP significantly reduced dopamine and its metabolites, including DOPAC and HVA in the striatum. Post-treatment with 10 and 20 mg/kg PCA markedly resisted the further reduction in the levels of DA and its metabolites. Next, we observed the number of TH-immunoreactive cells in SN using immunohistochemistry analysis. As shown in Figure 2B and 2C, the MPTP group revealed significantly fewer TH-positive cells than the control group in SN. However, post-treatment with 10 mg/kg and 20 mg/kg of PCA could significantly prevent this loss. To further confirm these results, the expression of TH protein was measured by western blot analysis. The results (Figure 2D) showed that TH protein levels were significantly lower in the MPTP group than controls, and post-treatment with PCA 10 mg/kg and 20 mg/kg PCA could attenuate MPTP-induced TH decrease. Furthermore, to identify neuronal degeneration in midbrain, Nissl staining and α-Syn levels were then detected in our study. Nissl staining results (Figure 3A, 3B) revealed that the number of Nissl-stained neurons in MPTP group was fewer than in the control group; while post-treatment with PCA significantly elevated the number of Nissl-stained neurons in MPTP-induced mice. As shown in Figure 3C, the expression levels of α-Syn in MPTP group were significantly increased compared with the control group, whereas treatment with PCA significantly inhibited MPTP-induced the increase of α-Syn. These results suggested that PCA protected against MPTP-induced dopaminergic neuronal loss.

### PCA regulated the expression levels of PLK2, p-GSK3β and Nrf2 in MPTP- intoxicated mice

**Figure 4:**
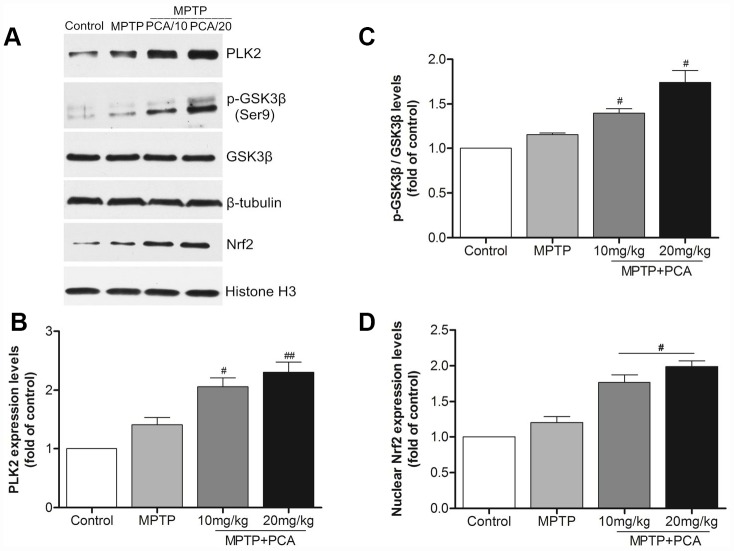
**PCA raised the expression levels of PLK2, p-GSK3β and nuclear Nrf2 in midbrain.** (**A**) Representative western blot bands of PLK2, p-GSK3β, GSK3β, Nrf2, tubulin and Histone H3 in each group. (**B**–**D**) Quantification of PLK2, p-GSK3β/GSK3β and nuclear Nrf2 levels in midbrain tissues. Data were expressed as mean ± S.D., n = 6; ^*^P< 0.01 *vs.* control group, ^#^*P*< 0.05 *vs.* MPTP group.

To examine the effects of PCA on the protein levels of PLK2, p-GSK3β and nuclear Nrf2, western blot analysis was performed at 24 h after the last administration of PCA. As shown in Figure 4, the protein levels of PLK2 as well as p-GSK3β and nuclear Nrf2 were slightly increased in MPTP intoxicated mice. With PCA administration, the protein levels of PLK2, p-GSK3β and Nrf2 were significantly increased when compared to the MPTP group. Our results also revealed that PCA (10 and 20 mg/kg) post-treatment had a dose-dependent effect on protein. These results indicate that the neuroprotective effects of PCA (10 and 20 mg/kg) appear to be mediated through the activation of the PLK2/p-GSK3β/Nrf2 signaling pathway *in vivo*.

### PCA reduced cytotoxicity and apoptosis in MPP^+^-incubated SH-SY5Y cells

**Figure 5:**
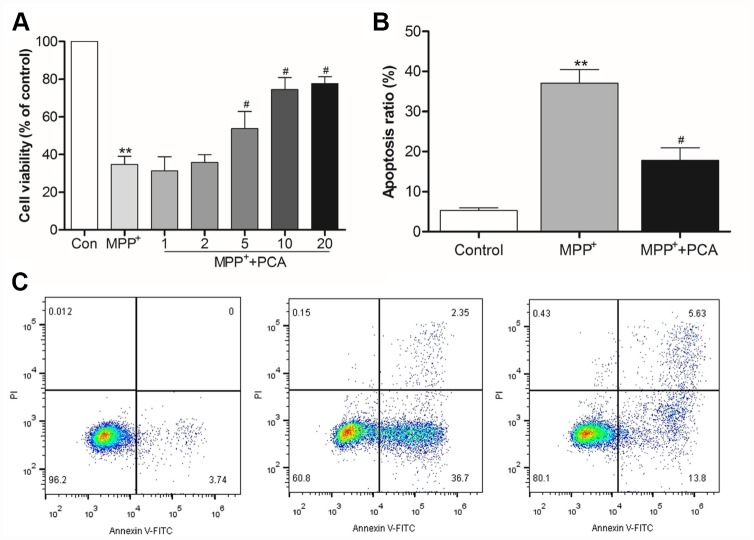
**PCA reduced cytotoxicity and apoptosis in MPP^+^-incubated SH-SY5Y cells.** (**A**) Histogram showing cell viability at different dosages of PCA. (**B**) Quantification analysis of apoptosis rate. (**C**) Representative pictures of flow cytometry for apoptosis in each group. Data were presented as mean ± S.D., n = 6; ^**^P< 0.01*vs.* control group; ^#^*P*< 0.05 *vs.* MPP^+^group.

The effect of PCA on the cytotoxicity of MPP^+^ in SH-SY5Y cells was investigated using MTT assay. As shown in Figure 5A, the measurements revealed a significant decrease in the viability of SH-SY5Y cells following exposure to 1mM of MPP^+^ for 24 h, while the cells treated with 5, 10, and 20 μM of PCA for 4 h significantly decreased MPP^+^-induced cytotoxicity. The results also revealed that PCA treatment exhibited concentration-dependent effects, but there was a similar ceiling effect once exceeding the dosage of 10 μM. Therefore, 10 μM of PCA was deemed to be the best effects in MPP^+^-induced cells. We then examined the MPP^+^-induced apoptosis of SH-SY5Y cells when were treated with 10 μM of PCA (Figure 5B, 5C). Incubation of SH-SY5Y cells with 1mM of MPP^+^ for 24 h significantly increased the percentage of apoptosis. Treatment with 10 μM of PCA for 4 h before MPP^+^ addition decreased the percentage of apoptosis, compared to that of the MPP^+^ group alone.

### PCA improved mitochondrial function and inhibited ROS production in MPP^+^-incubated SH-SY5Y cells

**Figure 6:**
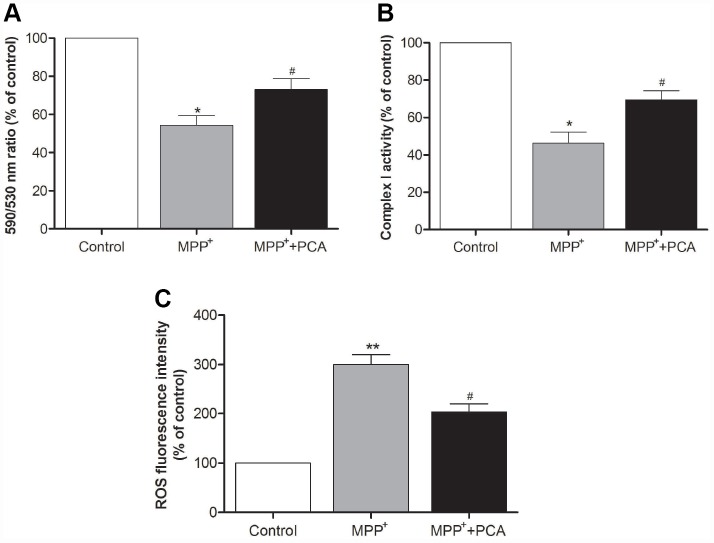
**PCA improved mitochondrial function and inhibited ROS production in MPP^+^-incubated SH-SY5Y cells.** (**A**) Mitochondrial membrane potential (MMP) was measured using the JC-1 probe through the 590 nm/530 nm fluorescence emission ratio. (**B**) Activity of mitochondrial complex I in each group. (**C**) Mitochondria-generated ROS levels in each group. Data were presented as mean ± S.D., n = 6; ^**^P< 0.01, ^*^P < 0.05 *vs.* control group; ^#^*P*< 0.05 *vs.* MPP^+^group.

To investigate whether PCA exerts protective effects on mitochondria function, mitochondrial membrane potential was evaluated with JC-1. Incubation of SH-SY5Ycells with 1 mM MPP^+^ for 24 h induced a significant reduction in the 590 nm/530 nm ratio when compared to control conditions, indicating that the MMP is severely reduced by the MPP^+^ treatment. Treatment with PCA prominently reversed the MPP^+^- induced loss of MMP (Figure 6A). Moreover, we further measured the activity of mitochondrial complex I. The result showed that PCA preserved the activity of mitochondrial complex I in MPP^+^-induced cells (Figure 6B). As shown in Figure 6C, ROS production in the MPP^+^ group markedly increased when compared with the control group. When treated with PCA, ROS production was significantly decreased as compared with the MPP^+^ group.

### PCA increased the expression of PLK2 and activated the p-GSK3β/Nrf2 pathway in MPP^+^-incubated SH-SY5Y cells

**Figure 7:**
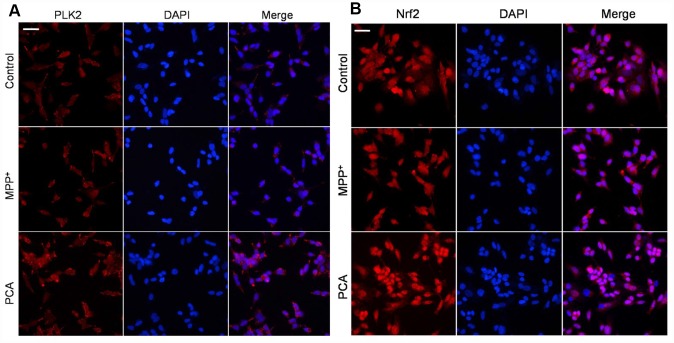
**PCA increased PLK2- and Nrf2-positive expression in MPP^+^-incubated SH-SY5Y cells.** (**A**) Representative immunofluorescent staining for PLK2 (bright red) and DAPI (blue). (**B**) Representative immunofluorescent pictures for Nrf2 (bright red) and DAPI (blue). Scale bar, 20 μm.

**Figure 8:**
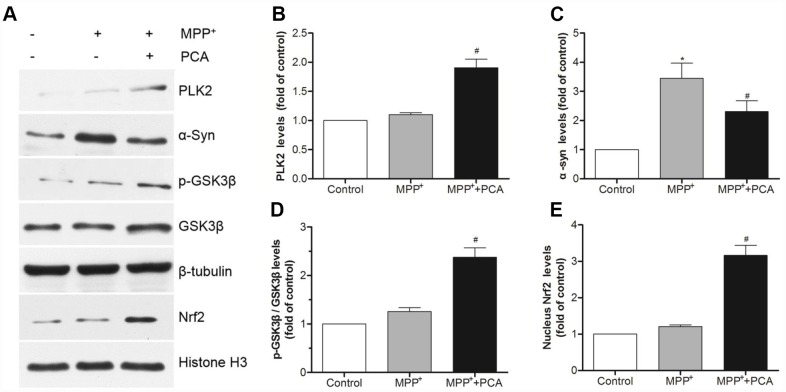
**PCA increased the expressions of PLK2 and activated p-GSK3β/Nrf2 pathway in MPP^+^-incubated SH-SY5Y cells.** (**A**) Representative western blot bands of PLK2, α-Syn, p-GSK3β, GSK3β, Nrf2, tubulin and Histone H3. (**B**–**E**) Quantification of PLK2, p-GSK3β/ GSK3β and nucleus Nrf2 levels in cells. Data were presented as mean ± S.D., n = 6; ^*^P < 0.05 *vs.* control group; ^#^*P*< 0.05 *vs.* MPP^+^group.

To further establish the role of PLK2 and Nrf2 in the neuroprotection of PCA in MPP^+^-incubated SH-SY5Y cells, immunofluorescence staining and western blot were performed respectively when PCA-treated cells were exposed to MPP^+^ incubation for 24 h. As shown in Figure 7, immunofluorescence staining revealed that the expression of PLK2- and Nrf2-positive cells increased less in the MPP^+^ group compared to that of the control group. Treatment with PCA increased the expressions of PLK2- and Nrf2-positive cells compared to the MPP^+^ group. Similar to results from western blot analysis, as shown in Figure 8, after SH-SY5Y cells were incubated with MPP^+^ for 24 h, PLK2, p-GSK3β, and nuclear Nrf2 expression did not show distinct changes, but the expression levels of α-Syn markedly increased. In contrast, PCA treatment remarkably promoted the PLK2, p-GSK3β, and nuclear Nrf2 expression as well as the reduction of α-Syn. The results indicated that PCA exerts neuroprotective effects by regulating PLK2, p-GSK3β and Nrf2 expression.

### The neuroprotection of PCA involved the PLK2/ GSK3β/Nrf2 pathway

**Figure 9:**
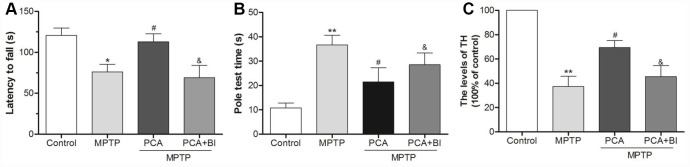
**BI2536 (BI) reversed the neuroprotection effect of PCA.** (**A**) Rotarod test in control, MPTP, PCA+MPTP and PCA+MPTP+BI group. (**B**) Pole test in each group. (**C**) TH levels from SN tissues were measured by ELISA. Data were expressed as mean ± S.D., n=6; ^*^P< 0.05, ^**^P< 0.01 vs. control group; ^#^*P*< 0.05 *vs.* MPTP group, ^&^*P*< 0.05 *vs.* PCA+MPTP group.

**Figure 10:**
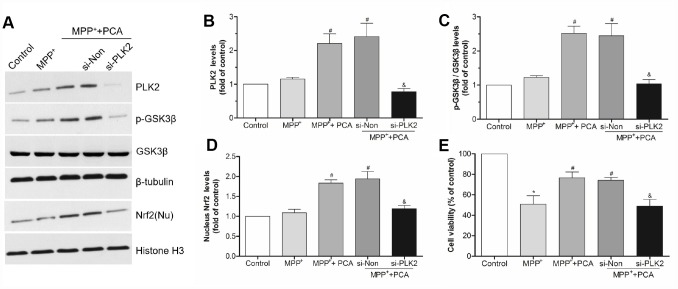
**PLK2 knockdown attenuated the protection of PCA.** (**A**) Representative western blot bands showing the expressions of PLK2, p-GSK3β and nucleus (Nu) Nrf2 when SH-SY5Y cells were transfected with PLK2 siRNA (si-PLK2) and Non-targeting siRNA (si-Non) for 72h. (**B**–**D**) The quantification of PLK2, p-GSK3β and nucleus Nrf2 levels in si-PLK2 group and the others groups. (**E**) Cell viability was measured by MTT assay. Data were presented as mean ± S.D., n = 6; ^*^P < 0.05 *vs.* control group; ^#^*P*< 0.05 *vs.* MPP^+^group; ^&^*P* < 0.05 *vs.* si-Non group.

**Figure 11:**
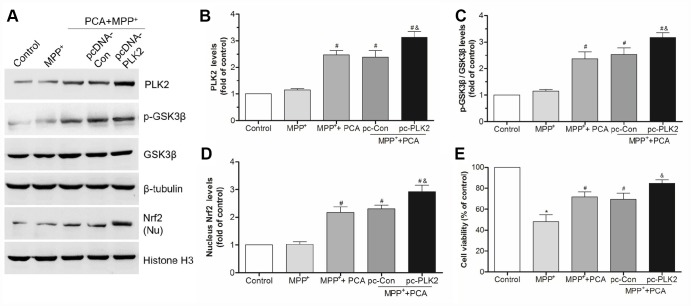
**Overexpression of PLK2 strengthened the protection of PCA.** (**A**) The bands of PLK2, p-GSK3β and nucleus Nrf2 after transfected with pcDNA3.1-PLK2 vector (pcDNA-PLK2, pc-PLK2) and the empty vector control (pcDNA-Con, pc-Con) for 48 h. (**B**–**D**) The quantification of PLK2, p-GSK3β/ GSK3β and nuclear Nrf2 levels in each group. (**E**) The cell viability in each group. Data were presented as mean ± S.D., n = 6; ^*^P < 0.05 *vs.* control group; ^#^*P*< 0.05 *vs.* MPP^+^group; ^&^*P* < 0.05 *vs.* pc-Con group.

**Figure 12:**
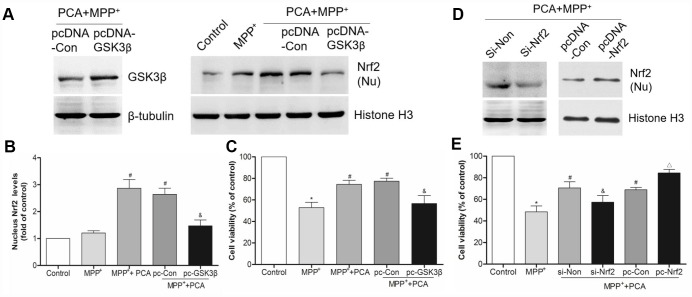
**GSK3β and Nrf2 were involved in PCA-induced protection.** (**A**) The bands of GSK3β and nucleus Nrf2 after transfected with pcDNA3.1-GSK3β expression vector. (**B**) Quantification of nucleus Nrf2 levels after transfected with pcDNA3.1-GSK3β vector. (**C**) Analysis of cell viability in each group. (**D**–**E**) The expression of nucleus Nrf2 and cell viability after transfected with siRNA-Nrf2 and pcDNA3.1-Nrf2 vector. Data were presented as mean ± S.D., n = 6; ^*^P < 0.05 *vs.* control group; ^#^*P*< 0.05 *vs.* MPP^+^group; ^&^*P* < 0.05 *vs.* si-Non group; ^△^*P* < 0.05 *vs.* pc-Con group.

To characterize the key role of PLK2 in PCA induced neuroprotection, BI2536 were used for blocking PLK2 activity and then evaluated whether the neuroprotection of PCA had been reversed. As shown in Figure 9, administration of BI2536 could abolished the improvement of behavioral deficits and resisted the increase of TH levels induced by PCA post-treatment in mice. To further verify that PLK2/p-GSK3β/Nrf2 pathway is indeed involved in the protection of PCA, the expressions of PLK2, GSK3β and Nrf2 were inhibited and enhanced by using siRNA and pcDNA 3.1(+) vector, respectively. As shown in Figure 10A–10D, we found that transfection with si-PLK2 significantly decreased the expressions of p-GSK3β and nuclear Nrf2 in PCA-treated cells following 24 h of MPP^+^ incubation compared to that of cells transfected with si-Non. Knockdown of PLK2, cell viability preservation provided by PCA was also markedly reversed (Figure 10E). Conversely, with PLK2 overexpression, the levels expression of p-GSK3β and nuclear Nrf2 were obviously increased (Figure 11A–11D), and the protection of PCA was enhanced (Figure 11E). We next investigated whether GSK3β could influence indirectly the protection of PCA. As shown in Figure 12A–12C, it has been observed that the overexpression of GSK3β can attenuate the nuclear translation of Nrf2 and PCA-induced protection. Moreover, the relationship between nuclear Nrf2 and the protection of PCA was also evaluated in cell experiment. we found that Nrf2 knockdown by siRNA significantly abolished the cyto-protection of PCA, while the overexpression of Nrf2 improved PCA-induced protection (Figure 12E). These results confirmed that PLK2/p-GSK3β/Nrf2 pathway is implicated with the neuroprotection of PCA.

## DISCUSSION

There remains a shortage of effective drugs for PD treatment, whereas the number of people being affected keeps increasing. PCA is a phenolic acid compound that has been verified to perform significant antioxidant effects. Although previous studies have shown that the MPTP-induced mice were orally pretreated with PCA for 14 days to protect against Parkinson’s disease [24], the present studies further certified that post-treatment of PCA can also exert significant neuroprotection in MPTP-induced mice by intraperitoneal injection for 5 days. Our data provided a powerful complement to the therapeutic effect of PCA against PD. Prominently, our studies propose a novel mechanism for neuroprotection in which PCA enhances PLK2 expression, as well elevates p-GSK3β and nuclear Nrf2 expression.

Neurotoxin MPTP has been the most valuable drug for inducing PD animal models [25]. MPP^+^, the active metabolite of MPTP, was formed through MPTP oxidation by monoamine oxidase-B *in vivo*, resulting in the injury of dopaminergic neurons. *In vitro* model usually used in PD research include PC12 and SH-SY5Y cells. PC12 is a cell line derived from a pheochromocytoma of the rat adrenal medulla. Upon exposure to nerve growth factor (NGF), PC12 cells can undergo neuronal differentiation. NGF-treated PC12 cells can release several neurotransmitters including dopamine, noradrenaline and acetylcholine. The human cell line SH-SY5Y is a subline of the SK-N-SH, which are isolated from a bone marrow biopsy with metastatic neuroblastoma. When stimulated with retinoic acid (RA), SH-SY5Y differentiates to dopaminergic neuron-like cells since it has been widely used as a PD cell model [26]. Moreover, SH-SY5Y neuroblastoma cells can be relatively easily genetically modified to mimic human disease-causing mutations to investigate their role in DA neuronal cellular model [27]. Therefore, the MPP^+^-induced SH-SY5Y cell injury model and MPTP-induced dopaminergic neuron loss in C57BL/6 mice were used in our present study to simulate PD. Our studies demonstrated that PCA effectively increased the duration time of mice on the rod and decreased the total time for climbing down the pole when compared with the MPTP-treated group. Moreover, PCA could also resist the decrease in the levels of DA and its metabolites in the striatum of MPTP-lesioned mice. The result suggested that the effect of PCA was closely associated with the protection of dopaminergic neurons against MPTP-induced neurotoxicity in the brain. TH, a key enzyme in dopamine biosynthesis, is closely related to the occurrence and development of PD [28]. To further demonstrate the effects of PCA on PD, immunohistochemistry and western blot analysis for TH protein were conducted. The data showed that PCA could increase TH-positive cells in SN compared with the MPTP treatment group, and PCA could also increase the expression of TH in midbrain. Moreover, Nissl staining and the detection of α-Syn levels also showed that PCA could increase the neuronal survival and reduce α-Syn accumulation. Similarly, *in vitro* studies showed that PCA could effectively protect against MPP^+^-induced cell injury in a concentration-dependent manner at 1-10 μM by increasing cell viability and reducing apoptosis. Interestingly, 20μM of PCA did not further obtain better results, and its efficacy has a ceiling effect in MPP^+^-incubated SH-SY5Y cells. The results suggested that PCA could be a promising agent of PD treatment.

Several theories have been suggested for the pathogenesis of PD, of which mitochondrial dysfunction and subsequent oxidative stress play dominant roles in PD pathogenesis [29, 30]. Mitochondrial dysfunction in PD could be evaluated by the detection of mitochondrial complex I activity and mitochondrial membrane potential change. It has been reported [31] that mitochondrial toxins, such as MPTP (MPP^+^), inhibit mitochondrial complex I of the electron transport chain, leading to an increase in the production of ROS and mitochondrial oxidative stress, which causes selective degeneration of the dopaminergic neurons in SN and α-Syn accumulation. It is well known that α-Syn is the most characteristic hallmark of PD. Previous evidence suggested that the interaction of mitochondria with α-Syn could exert an important effect in the PD-associated mitochondrial dysfunction [32]. The aggregation of α-Syn within mitochondria has been shown to inhibit mitochondrial complex I activity [33, 34]. In postmortem PD brains, the increased accumulation of α-Syn within the mitochondria has been found in the regions of the SN and striatum, and this study has further demonstrated the interaction of α-Syn with complex I [35]. Massed α-Syn has been also reported to suppress mitochondrial complex I activity, resulting in the damage of ATP synthesis and mitochondrial respiration [36]. It has been observed that mitochondrial dysfunction induced by α-Syn could cause the increased production of ROS [37, 38]. Furthermore, over-expression of wild or mutant α-Syn in SH-SY5Y cells has been shown to increase the intracellular level of ROS [39]. Consistent with previous research, our studies have demonstrated that complex I activity and MMP were significantly decreased in MPP^+^-incubated SH-SY5Y cells with α-Syn accumulation. However, treatment with PCA could reverse the MPP^+^-induced decreases. In addition, ROS levels produced by mitochondria were obviously inhibited when MMP^+^-incubated cells were pretreated with PCA for 4 h. These data indicated that PCA can repair mitochondrial dysfunction and prevent mitochondrial ROS-induced oxidative damage.

PLKs belong to a family of serine/threonine kinases that perform multiple functions in the cell cycle and growth factor-mediated neuronal differentiation [40]. Noteworthily, PLK2, as a subtype of the PLKs, have been reported to have high expression in cells, and it is necessary for cell survival with oxidative stress and mitochondrial dysfunction. PLK2 has vital functions in postmitotic neurons during the process of neurodegenerative disease, such as PD [41]. In the PD model, PLK2 has been shown to phosphorylate and facilitate selective autophagic clearance of α-Syn, suggesting a viable target for PD treatment [42]. Recent research has also reported that PLK2 can inhibit α-Syn expression levels by regulating α-Syn mRNA production in mice and in all tested cell types, including the SH-SY5Y cells line [43]. In addition, PLK2 also plays important roles in sustaining dendritic spine stability and regulating excitatory glutaminergic synaptic connections [44], and the elevated levels of phosphorylated α-Syn in the cerebrospinal fluid of PD patients could be a biomarker of PLK2 activation as an antioxidant response [45]. Another possibility is that the abnormal accumulation of α-Syn may contribute to PD through suppressing PLK2 activation and subsequently destroying redox homeostasis [46]. Previous studies have determined that Ser-9 of GSK3β is candidate substrate of PLK2 [47]. GSK3β is plentiful in the central nervous system, particularly in neurons [48]. Activation of GSK3β has been suggested to be related to the neurodegenerative disorders [49]. Regulation of GSK3β activity is critically dependent on the phosphorylation state of its Ser9 [50]. Phosphorylation of GSK3β at the Ser-9 residue is a competitive pseudo-substrate that auto-inhibits its kinase activity. Nrf2 is targeted by GSK3β, whereas oxidative stress can induce GSK3β activation and subsequently down-regulate the Nrf2 antioxidant-related genes, therefore restricting the cellular antioxidant capacity [51, 52]. Thus, the increased PLK2 expression should inhibit the GSK3β activity by up-regulating its phosphorylation, leading to Nrf2 translocation into the cell nucleus and then transactivation of various downstream antioxidant genes. In our current studies, we have disclosed that the levels of PLK2 both in MPTP induced-mice and in MPP^+^ incubated cell were obviously increased by PCA treatment, as well as the elevated levels of p-GSK3β (Ser9) and enhanced nuclear Nrf2 expression. BI 2536 is a pan-PLK inhibitor which has a high selectivity for PLK2. Inhibition of PLK2 with BI2536 could reverse the neuroprotection of PCA *in vivo*. The result suggested that PLK2 plays a key role in PCA-induced neuroprotection. To further confirm PLK2 and its downstream proteins involved in the neuroprotection of PCA, the expressions of PLK2, GSK3β and Nrf2 were also regulated by transfection with siRNAs and pcDNA3.1(+) *in vitro*. Our studies showed that the protection of PCA is positively correlated with the expression levels of PLK2 and nuclear Nrf2, but negatively correlated with GSK3β levels. These results further demonstrated that PCA mediates PLK2 expression, which up-regulates GSK3β phosphorylation and Nrf2 nuclear translocation, resulting in neuroprotection against PD injury.

Together, our findings elucidated that PCA effectively exerts neuroprotection in MPTP or MPP^+^-induced PD models through improvement mitochondrial dysfunction and attenuation of oxidative stress injury, and its neuroprotection is involved in PLK2/p-GSK3β/Nrf2 pathway. These results help to better understand the molecular mechanisms of the neuroprotective effect of PCA and may support compelling evidence in the search for more potent pharmacological agents to protect against mitochondrial function and oxidative stress for treating PD.

## MATERIALS AND METHODS

### Drugs and chemicals

PCA (CAS 139-85-5, purity≥98%) was obtained from the Pufei De Biotech Co., Ltd. (Chengdu, China). Fetal bovine serum (FBS), Dulbecco’s modified Eagle’s medium (DMEM), Ham’s F12 and trypsin were supplied by Gibco (Life Technologies, Grand Island, NY, USA). 1-Methyl-4-phenyl-1, 2, 3, 6-tetrahydropyridinehydrochloride (MPTP), 1-methyl-4-phenylpyridinium iodide (MPP^+^) and all-trans retinoic acid (RA) were obtained from Aladdin-e (Shanghai, China). Mitochondrial membrane potential (MPP), ROS, MTT and Annexin V-FITC assay kits were provided by Beyotime Institute of Biotechnology (Nantong, China). The protein extraction kits, BCA protein assay kits, and mitochondrial complex I assay kits were purchased from Nanjing Jian Cheng Bioengineering Institute (Nanjing, China). Lipofectamine 3000 transfection reagents were obtained from Invitrogen Life Technologies (Carlsbad, CA, USA). Antibodies of PLK2, tyrosine hydroxylase (TH), and α-synuclein (α-Syn) were purchased from Abcam PLC (Abcam, Cambridge, UK). Anti-Nrf2, anti-p-GSK3β (Ser9) and GSK3 β antibodies were supplied by Cell Signaling Technology, Inc. (CST, Danvers, MA, USA). BI2536, an inhibitor, was provided by Target Mol (Boston, MA, USA). All other chemicals were obtained from commercial corporations in the highest grade available.

### Animals and treatment

Male C57BL/6 mice (age, 9-10 weeks; weight, 20–25g) were provided by the Experimental Animal Center of the Fourth Military Medical University. The animals were kept at 23±2 °C with 12 h light/dark cycles and fed on standard chow diet and water *ad libitum*. All experimental protocols were approved by the Ethics Committee for Animal Experimentation of the Fourth Military Medical University (Xi’an, China).

Mice were randomly divided into four groups: control group; MPTP group; MPTP + PCA 10 mg/kg group; MPTP + PCA 20 mg/kg group. MPTP-induced PD model was established according to previously described methods [53, 54]. All mice except the control group were administered an intraperitoneal injection of MPTP in saline at a dosage of 30 mg/kg/d for 7 consecutive days. Mice in control group were given 10 mL/kg/d saline for 7 days. After the last MPTP injection, mice were then treated with PCA (dissolved in sterile water) or the same volume of water once a day for 5 days by intraperitoneal administration. At 24 h after the last administration of PCA or water, behavioral (rotarod and pole) test, neurochemical measurement, Nissl staining, immunohistochemistry staining and western blot analysis were performed.

### Rotarod test

The rotarod test was used to measure sports coordination in mice as previously described [55]. Firstly, mice were trained on a stationary rod (2.5 cm diameter) for 30 s and then trained at a constant velocity of 16 rpm for a period of 180 s. Sixty minutes after the last training, a mouse was placed on the rod, and the time taken until the mouse fell from the rod was recorded. The average time of three trials was calculated for statistical analyses.

### Pole test

The pole test was used to analyze the degree of bradykinesia and was carried out as previously described [56]. Briefly, a mouse was placed facing upward near the top of a wooden pole with a rough surface (10 mm in diameter and 55 cm in height). The mouse was placed on the top of the pole facing head up. The total time for each animal to reach the base of the pole was recorded. The test of each mouse was performed three times at 10-min intervals, and the average time was calculated for statistical analyses.

### Measurement of dopamineand its metabolitelevels

Levels of dopamine and its metabolites 3, 4-dihydroxyphenylacetic acid (DOPAC) and homovanillic acid (HVA) in striatum were quantified by high-performance liquid chromatography (HPLC) with a photodiode array detector (SPD-M20A, Shimadzu, Japan) as previously reported [57]. Briefly, striatum was dissected on an ice-chilled plate and stored at -80°C until analysis. Striatal tissue was homogenized and then centrifuged at 15,000 *g* for 20 min at 4°C. The supernatant was filtered through a 0.22-μm membrane filter, and a 10 μl aliquot of the sample was injected onto the HPLC column for assay. The mobile phase (0.1M citrate buffer, 1 mM 1-octanesulfonic acid, 0.02 mM EDTA, 10% MeOH, PH 4.5) was delivered at a rate of 1 mL/min. The value is expressed as a percentage with the control group as 100%.

### Sample preparation

In Nissl staining and immunochemistry analysis experiments, mice were firstly infused with 0.1M of phosphate buffered saline (PBS) to wash off the blood and then washed with freshly prepared 4% (w/v) paraformaldehyde in 0.1 M of PBS (pH=7.4). Subsequently, the brain was removed and post-fixed in 4% paraformaldehyde for 12-24 h. Brain blocks were embedded in paraffin after dehydration and cut into 6 μm coronal sections. Sections were used for Nissl and immunochemistry staining. For western blot analysis, the midbrain tissues containing SN were quickly dissected on ice. Samples were stored at 80°C for usage.

### Nissl staining

Nissl staining was performed according to the manufacturer’s instruction. Sections were dried overnight at room temperature and stained using 0.1% cresyl violet solution for 10-15 min. Sections were then dehydrated by 70% and 95% ethyl alcohol. Lastly, sections were treated by xylene for clearing tissues and mounted by DPX mounting medium. The sample images were inspected using an Olympus IX71 microscope.

### Immunohistochemistry of TH

Immunohistochemistry was conducted as previously reported [58]. Sections were routinely deparaffinized and rehydrated and then incubated with 3% H_2_O_2_ for 15 min, followed by 10% normal goat serum to inhibit nonspecific binding of the antibodies. Sections were incubated with the primary antibodies of TH at 4°C overnight, followed by incubation with a biotinylated secondary antibody for 1 h and horseradish peroxidase (HRP)-streptavidin for 30 minutes at room temperature. Sections were finally developed with diaminobenzidine for 5-10 min at room temperature and counterstained with hematoxylin. All sections were analyzed using an inverted microscope (IX71; Olympus Corporation, Tokyo, Japan). Immuno-positive cells were quantified using software.

### Cell culture and treatment

Human neuroblastoma SH-SY5Y cells were provided by the American Type Culture Collection (ATCC, Manassas, VA, USA). Cells were cultured in Ham’s F12 and DMEM-F12 medium supplemented with 10% (v/v) FBS and 100 U/ml penicillin/streptomycin. Cultures were kept in a humidified incubator at 37°C in an atmosphere of 5% CO_2_ and 95% air. 10 μM retinoic acid (RA) was added to the medium for stimulating the cells to differentiate. The medium was replaced once every 2 days.

For cell viability assays, differentiated SH-SY5Y cells were treated for 4 h with PCA at different concentrations (1, 2, 5, 10, 20 μM) and then incubated with 1mM of MPP^+^ for 24 h. In other experiments, cells were treated with the optimal concentration of PCA (10μM) and MPP^+^(1mM) for the same period.

### Cell viability assay

Cell viability was determined using an MTT assay. Briefly, cells were seeded in 96-well plates. After incubation with MPP^+^, MTT solution (5mg/ml) was added into each well for 4 h at 37°C. The precipitated formazan crystal was dissolved in dimethyl sulfoxide (DMSO), and absorbance was measured at 570 nm using a microplate reader. Cell viability was expressed as a percentage with the control group as 100%.

### Flow cytometry

Cell apoptosis was measured by flow cytometry. Briefly, cells were harvested and washed with PBS after 24 h incubation of MPP^+^. The cells were resuspended and incubated with 5 μL Annexin V-FITC and 5 μL propidium iodide. Apoptosis was quantified by flow cytometer. The apoptosis percentage was calculated including early apoptosis (Annexin V^+^/PI^-^) and late apoptosis (Annexin V^+^/PI^+^). Experiments were repeated three times to ensure reproducibility.

### Measurement of mitochondrial membrane potential

MMP was evaluated by an MMP assay kit with JC-1 according to the manufacturer’s instructions. JC-1 is a dual-emission membrane potential-sensitive probe that exists as a green fluorescent monomer at a low MMP and forms aggregates with red / orange fluorescence at a high MMP. Briefly, cells cultured in 6-well plates were washed twice with PBS, and 2.5μM JC-1 was added for 20 min at 37°C in the dark. Following washing with PBS, the proportion of aggregated versus monomeric JC-1 probe was quantified by measuring the ratio of fluorescence emissions at 590 nm (red) over 530 nm (green) with a flow cytometer.

### Determination of mitochondrial complex I activity

Mitochondrial complex I activity was measured using the Mitochondrial Complex I Activity Assay Kit according to the manufacturer’s protocol. Cells were firstly disrupted by one freezing in liquid nitrogen and rapidly thawed at 37°C. Fifty micrograms of mitochondrial protein were loaded into the well of a microplate coated with complex I capture antibody, and incubated for 1 h at 37°C. Complex I activity was determined by measuring the oxidation of nicotinamide adenine dinucleotide (NADH) to NAD^+^, which was observed as a reduction in the dye and a corresponding increase in absorbance at 450 nm. Each sample was tested in triplicate and the activity is expressed as the change in absorbance per minute per microgram protein.

### ROS assay

Intracellular ROS levels were determined using 2', 7'-dichlorofluorescein diacetate (DCFH-DA) staining as described previously [59]. Incubation of MPP^+^ for 24 h, cells were washed twice with PBS and continually incubated with 10 μM of DCFH-DA at 37°C for 30 min according to the manufacturer’s instructions. The fluorescence was read at 485 nm for excitation and 525 nm for emission with a fluorescence plate reader. The ROS production was expressed as percentage in fluorescence relative to the control group.

### Immunofluorescence staining

After 24 h of incubation of MPP^+^, SH-SY5Y cells were fixed by 4% paraformaldehyde for 10 min and permeabilized with PBS containing 0.1 % Triton X-100 for 10 min. cells were blocked in 10% goat serum and incubated overnight with primary antibodies against PLK2 and Nrf2 at 4°C. Cells were washed with PBS and then incubated with fluorescent secondary antibody (conjugated with Alexa Fluor®594 for 1 h. Subsequently, cells were counterstained with 4′,6-diamidino-2-phenylindole (DAPI) and imaged with a fluorescence microscope. Fluorescent intensities were measured using Image J software.

### PLK2 inhibitor treatment and cell transfection

*In vivo* experiments, mice were given 30 mg/kg dose of BI2536 or 5mL/kg vehicle by intravenous injection one hour before receiving PCA (20mg/kg) daily treatment to block PLK2 activity. Behavioral test and the levels of TH were measured at 24 h after the last administration of PCA.

*In vitro* experiments, the small interfering RNAs (siRNAs) targeting PLK2, Nrf2 and non-targeting (si-Non) were designed and synthesized by Wuhan Servicebio Technology Co., Ltd (Wuhan, China). The over-expression plasmids of pcDNA3.1-PLK2, GSK3β, Nrf2 and their control plasmids were prepared by Fenghbio Co. Ltd. (Changsha, China). Transfection was performed by using Lipofectamine according to the manufacturer’s instructions. At 48-72 h after transfection, cells were treated with 10 μM of PCA and then incubated with 1mM of MPP^+^ for 24 h. Cell samples were collected for MTT assay.

### Western blot analysis

Western blot analysis was performed according to the standard protocols. Total and nuclear protein from SH-SY5Y cells and midbrain tissues were extracted with homogenization buffer containing a protease inhibitor. The extracted protein concentrations were determined using the BCA protein assay kit. Equal amounts of protein were separated by 10% sodium dodecyl sulfate-polyacrylamide gel electrophoresis (SDS-PAGE) and subsequently transferred to polyvinylidene difluoride (PVDF) membranes. The membranes were blocked with 5% BSA in tris-buffered saline with Tween (TBST) and incubated overnight at 4°C with primary antibodies to anti-TH, anti-α-Syn, anti-PLK2, anti-GSK3β, anti-p-GSK3β, β-tubulin, anti-Nrf2, and histone H3. Subsequently, the membranes were incubated with HRP-conjugated secondary antibody for 2 h at 37°C. The blots were visualized using the enhanced chemiluminescence method (ECL Kit; Pierce, Rockford, IL, USA), and the bands were scanned and analyzed with Quantity One image analysis software (Bio-Rad Laboratories).

### Statistical analysis

All statistical analyses in this study were performed using GraphPad Prism 5 for Windows. Data were expressed as mean ± standard deviation (S.D.) and were compared using a one-way analysis of variance (ANOVA) followed by Tukey’s multiple-comparison test. A value of *P*< 0.05 was considered as statistically significant.
